# The MtZ Strain: Molecular Characteristics and Outbreak Investigation of the Most Successful *Mycobacterium tuberculosis* Strain in Aragon Using Whole-Genome Sequencing

**DOI:** 10.3389/fcimb.2022.887134

**Published:** 2022-05-24

**Authors:** Jessica Comín, Jan Madacki, Isabel Rabanaque, María Zúñiga-Antón, Daniel Ibarz, Alberto Cebollada, Jesús Viñuelas, Luis Torres, Juan Sahagún, Christophe Klopp, Jesús Gonzalo-Asensio, Roland Brosch, María-José Iglesias, Sofía Samper

**Affiliations:** ^1^ Grupo de Genética de Micobacterias, Instituto Aragonés de Ciencias de la Salud, Zaragoza, Spain; ^2^ Unit for Integrated Mycobacterial Pathogenomics, Institut Pasteur, Université de Paris, CNRS UMR 3525, Paris, France; ^3^ Departamento de Geografía y Ordenación del Territorio, Universidad de Zaragoza, Zaragoza, Spain; ^4^ Instituto Universitario de Investigación en Ciencias Ambientales de Aragón, Zaragoza, Spain; ^5^ Fundación Instituto de Investigación Sanitaria (IIS) Aragón, Zaragoza, Spain; ^6^ Grupo de Genética de Micobacterias, Facultad de Medicina, Universidad de Zaragoza, Zaragoza, Spain; ^7^ Unidad de Biocomputación, Instituto Aragonés de Ciencias de la Salud, Zaragoza, Spain; ^8^ Hospital Universitario Miguel Servet, Zaragoza, Spain; ^9^ Grupo de Estudio de Infecciones por Micobacterias (GEIM), Sociedad Española de Enfermedades Infecciosas y Microbiología Clínica, Madrid, Spain; ^10^ Hospital San Jorge, Huesca, Spain; ^11^ Hospital Clínico Universitario Lozano Blesa, Zaragoza, Spain; ^12^ MIAT INRA, Castanet-Tolosan, France; ^13^ Centro de Investigación Biomédica en Red (CIBER) de Enfermedades Respiratorias, Madrid, Spain

**Keywords:** tuberculosis outbreak, molecular epidemiology, tuberculosis, WGS, tuberculosis virulence

## Abstract

Since 2004, a tuberculosis surveillance protocol has been carried out in Aragon, thereby managing to detect all tuberculosis outbreaks that take place in the community. The largest outbreak was caused by a strain named *Mycobacterium tuberculosis Zaragoza* (MtZ), causing 242 cases as of 2020. The main objective of this work was to analyze this outbreak and the molecular characteristics of this successful strain that could be related to its greater transmission. To do this, we first applied whole-genome sequencing to 57 of the isolates. This revealed two principal transmission clusters and six subclusters arising from them. The MtZ strain belongs to L4.8 and had eight specific single nucleotide polymorphisms (SNPs) in genes considered to be virulence factors [*ptpA*, *mc3D*, *mc3F*, *VapB41*, *pks15* (two SNPs), *virS*, and *VapC50*]. Second, a transcriptomic study was carried out to better understand the multiple IS*6110* copies present in its genome. This allowed us to observe three effects of IS*6110*: the disruption of the gene in which the IS*6110* is inserted (*desA3*), the overexpression of a gene (*ppe38*), and the absence of transcription of genes (*cut1:Rv1765c*) due to the recombination of two IS*6110* copies. Finally, because of the disruption of *ppe38* and *ppe71* genes by an IS*6110*, a study of PE_PGRS secretion was carried out, showing that MtZ secretes these factors in higher amounts than the reference strain, thereby differing from the hypervirulent phenotype described for the Beijing strains. In conclusion, MtZ consists of several SNPs in genes related to virulence, pathogenesis, and survival, as well as other genomic polymorphisms, which may be implicated in its success among our population.

## Introduction

Airborne tuberculosis (TB) has existed alongside humanity since the beginning of civilization, being first recorded among Egyptian mummies dating back to 2,400 BC (Morse et al., 1964). In 2019, an estimated 10 million people fell ill with TB, causing 1.2 million deaths (World Health Organization, 2020). While COVID-19 surpassed TB as the world’s leading deadly infectious disease in 2020, TB remains second. *Mycobacterium tuberculosis* (MTB) is the causative pathogen, which usually produces respiratory disease but can also cause extrapulmonary disease (García-Rodríguez et al., 2011).

Traditionally, restriction fragment length polymorphism (RFLP), spoligotyping, and mycobacterial interspersed repetitive units-variable number of tandem repeats (MIRU-VNTR) have been used to investigate outbreaks due to its value in interpreting transmission dynamics (Borgdorff et al., 2000; Gonzalo-Asensio et al., 2018; Iglesias et al., 2020). The development of whole-genome sequencing (WGS) technologies marked a milestone in outbreak investigation, as it offers unsurpassed resolution power (Nikolayevskyy et al., 2019). Moreover, WGS has become an affordable technique, thus being proposed as a replacement for previous molecular typing techniques (Cirillo et al., 2016). However, its potential to elucidate the direction of transmission is still uncertain (Hatherell et al., 2016).

A TB molecular surveillance program has been carried out in the Autonomous Community of Aragon, in the north of Spain, since 2004. All MTB cases were genotyped using IS*6110-*RFLP and spoligotyping, and recently, WGS has been applied to relevant isolates. In a previous study for 2001–2004, a cluster of 85 cases was discovered (López-Calleja et al., 2007), the largest of the period. Subsequently, this strain was named *M. tuberculosis Zaragoza* (MtZ) and was classified as belonging to the principal genetic group 3 (López-Calleja et al., 2009). A study of the MtZ strain in terms of fitness demonstrated its high virulence in a low-dose aerosol and intravenous *in-vivo* infection model (Caceres et al., 2012). Some epidemiological links were found among several cases, but the virulence mechanisms that allowed this strain to spread affecting 242 cases until today were unknown.

The aims of this work were to complete the study of this outbreak, the largest in our region since our records began in 1993, by means of WGS and to determine the molecular characteristics that could be responsible for the diffusion of the MtZ strain.

## Materials and Methods

### Clinical Samples and Cases

Aragon has three capital provinces, including Zaragoza province where almost three-quarters of the Aragonese population lives, the majority of them in Zaragoza City. The microbiologists working in the mycobacterial units were the professionals in charge of encoding the tuberculosis strains (strain code) that arrived at the microbiology laboratories of Aragon’s hospitals. These microbiologists carried out the drug susceptibility tests for all the isolates in their laboratory routine. In our laboratory, a nurse determined the new TB cases and assigned the four-digit code for each case. It is necessary to have controlled the repeated cases. The strain code was the key field linked to the necessary variables for the study. No person from our research laboratory was involved in this process. All data remained anonymous. Our regional ethical committee (Comité de Ética de la Investigación de la Comunidad Autónoma de Aragón, Record No. 20/2018) approved this methodology, detailed in the 18/0336 project.

Since 2004, all *M. tuberculosis* isolates have been genotyped as part of a surveillance protocol, and the DNA remains frozen at −20°C. All MtZ cases were selected based on the identical IS*6110-*RFLP pattern (12 bands) of their isolates, analyzed by BioNumerics software (v7.6, Applied Maths, Kortrijk, Belgium). There were some exceptions, previously described by Millán-Lou et al. in 2015 (Isabel Millan-Lou et al., 2013) as evolved isolates with an extra or a lost band. Two cases were identified in Aragon from 1993 to 1995, and 240 cases were identified from January 2001 to December 2020. From all the samples with available DNA, 57 were sequenced using WGS: one from 1995 (considered case 0 in this study), 15 from 2001, two from 2002, two from 2003, three from 2004, three from 2006, three from 2007, two from 2009, four from 2010, three from 2011, three from 2012, one from 2013, two from 2015, two from 2016, three from 2017, five from 2018, and three from 2019.

### DNA Extraction and Genotyping

The DNA of the MtZ isolates, stored at −20°C until sequencing, was extracted from bacterial growth in solid media cultures at the time of diagnosis of the cases using the cetrimonium bromide method previously described (van Soolingen et al., 1994). IS*6110-*RFLP and spoligotyping were performed for all the isolates as previously described (Van Embden et al., 1993; Kamerbeek et al., 1997).

### WGS

Illumina sequencing (Nextera Flex, San Diego, USA) was applied using the manufacturer’s instructions for 43 samples. Ion Torrent technology was used on 14 samples according to the manufacturer’s instructions. After sequencing, millions of sequences contained in the fastQ files were mapped against the reference strain H37Rv (NC_000962.3) in order to obtain the Binary Aligned Map (BAM) and Variant Call Format (VCF) files, used for the single nucleotide polymorphism (SNP) study. The depth of the sequences was at least 30× and the average coverage was around 98%.

### Lineage Identification Using WGS

For lineage identification, the SNP classification established by Coll et al. in 2014 (Coll et al., 2014) was applied. This classification associates one strain to one lineage based on several representative SNPs of each TB lineage.

### Cluster Classification Using PCR

We designed three pairs of primers to be able to identify which main cluster the non-sequenced isolates of interest belonged to: deoA-F (CGACAAGTCACCTCCCTG) and deoA-R (GAGATGAACTGCCCGCT) for the SNP at point 3702632 (original CLS), dacB1-F (GCGCACCTGCTCGACTAC) and dacB1-R (CTAGTGCTGCGGCCG) for the SNP at point 3716874 (CLS-2), and PE12-F (TGGCCGTTTCGATATTGG) and PE12-R (GTCGAACCGTGGGTGC) for the SNP at point 1301938 (CLS-1).

### Bioinformatics for the Study of the Genomes

From the fastQ files obtained after sequencing, the BAM and VCF files were produced. These files were studied using the Integrative Genomics Viewer software [IGV, from the Broad Institute (Robinson et al., 2011)] to search antibiotic resistances and to compare the MtZ strain with other L4.8 strains we had sequenced for another study. Tuberculist (http://genolist.pasteur.fr/TubercuList/), Mycobrowser (https://mycobrowser.epfl.ch/) and UniProtKB (https://www.uniprot.org/uniprot/) websites were used to find information about the genes and proteins with interesting SNPs. We used GeneWise (https://www.ebi.ac.uk/Tools/psa/genewise/) to determine if the SNP was synonymous or non-synonymous and PROVEAN (http://provean.jcvi.org/seq_submit.php) to determine the effect of the non-synonymous SNPs (neutral or deleterious).

The fastQ files were also uploaded into BioNumerics software, where the phylogenetic trees were constructed using the UPGMA method. The program mapped the sequences against H37Rv and found the SNPs between the different isolates. SNPs in genes related to resistance were also determined. For greater accuracy, strict SNP filtering that removed positions with at least one ambiguous or unreliable base, gaps (maximum frequency 1%), non-discriminatory positions, and *ppe* and *pgrs* genes was applied. The retained SNP positions had a minimum of 5× coverage and the minimum distance between SNPs was at least 12 base pairs (bp). As the program was not able to compare sequences obtained by different sequencing platforms, SNPs were eye reviewed in both different phylogenetic trees constructed for the Illumina and Ion Torrent sequences to detect the shared SNPs.

### Geodatabase and Map Design

The location (street and number) is available for each MtZ case, and therefore, its assignment to a basic health zone (BHZ) is possible. This allowed the development of a geodatabase that would facilitate cross-referencing with sociodemographic data and its analysis. With this aim, we designed two maps to draw the spatial distribution of TB cases in the city of Zaragoza from 2001 to 2020 together with the two cases identified in the 1993–1995 period. Both maps used the BHZ of the city of Zaragoza as a spatial base, represented by polygons in a vector model of 133 elements. The coordinate system was ETRS 1989 UTM, Zone 30N. Cartography was implemented in a Geographic Information System (ESRI ArcGIS 10.7 software), where we prepared the base map and built the geodatabase. The first map had a multivariate design representing two variables: the number of cases and the TB rate per 100,000 population, calculated using the total number of cases due to the MtZ strain in each neighborhood taking into account the population of each BHZ in 2019. This was represented by color hue and lightness, and the number of cases was represented by size. Establishing spatial patterns in the first variable (TB rate) was based on a sequential scheme (yellow to brown) suited to ordered data that progress from high to low. The number of cases was represented using a graduated symbol system, where data were sorted into ranges to which a symbol size was assigned to represent the class mark. The second map showed the distribution of cases by CLS using a binary color scheme (orange–gray) showing nominal differences divided into two categories (presence–absence).

### Transcriptomic Analysis

With the objective of analyzing the effect of the IS*6110* in the *M. tuberculosis* genome, we studied three different MtZ isolates having the same genome background but carrying one different IS copy (case 0, the original strain with 12 IS*6110* bands; case 129, with an extra IS*6110* located in *dnaA:dnaN*; and case 241, with one less IS*6110* due to recombination around the DR region). They were cultured and sent to the STAB-VIDA Company (Caparica, Portugal, https://www.stabvida.com) for transcriptomic analysis. The library construction of cDNA molecules was carried out using a Ribosomal Depletion Library Preparation Kit. The generated DNA fragments were sequenced in the Illumina NovaSeq platform, using 150-bp paired-end sequencing reads. The analysis of the generated raw sequence data was carried out using CLC Genomics Workbench 12.0.3. The high-quality sequencing reads were mapped against the reference genome *M. tuberculosis* H37Rv (NC_000962.3). For the transcriptomic study, we used Integrative Genome Browser (IGB) software (Freese et al., 2016).

### PE_PGRS Secretion Analysis

One isolate of MtZ (MS 387, case 0) and H37Rv as a control were grown for protein extraction in 7H9-0.05% Tween-80 supplemented with dextrose, NaCl, and catalase to avoid albumin contamination in the secreted fraction until an optical density of 0.6–0.8 at 37°C was reached and then were pelleted. Supernatant fraction and whole-cell protein were extracted following the protocol described by Pérez et al. (2020). Secretion analysis was performed as described previously (Ates et al., 2018; Madacki et al., 2021). Briefly, the whole-cell protein and supernatant fractions were loaded on a NuPage 10% Bis-Tris gel (Thermo Fisher, Waltham, USA), followed by a dry Western blot transfer onto a nitrocellulose membrane (iBlot, Thermo Fisher). The membrane was incubated with anti-PGRS primary antibody 7C4.1F7 (1:2,000) (the antibody-producing clone was a gift from M. J. Brennan, Aeras, Rockville, MD, USA, and the purified antibody was a gift from W. Bitter, Amsterdam UMC, Amsterdam, the Netherlands) and a horseradish peroxidase (HRP)-conjugated IgG anti-mouse secondary antibody (Amersham) (1:5,000). As a loading control, anti-SigA (1:5,000) primary antibody (a gift from I. Rosenkrands, Statens Serum Institut, Copenhagen, Denmark) followed by HRP-conjugated IgG anti-rabbit secondary antibody (Amersham) (1:5,000) was used.

## Results

The MtZ strain was first observed in an epidemiological/genomic study (López-Calleja et al., 2007) carried out in the Aragon region, which produced the largest outbreak ever recorded there. The RFLP pattern showed 12 bands and a unique spoligotype, defined as unknown in the SITVIT2 database (http://www.pasteur-guadeloupe.fr:8081/SITVIT2/description.jsp). Its IS*6110* locations were studied by Millán-Lou et al. in 2013 (Isabel Millan-Lou et al., 2013). At that time, WGS was not developed enough for routine application in laboratories; therefore, the molecular characterization was not exhaustively studied. Now, we can observe its molecular properties in much more detail in order to find possible explanations for the high incidence of this strain in the local population of Aragon.

### MtZ Genomic Characterization

Using the phylogenetic SNP classification established by Coll et al. in 2014 (Coll et al., 2014), we concluded that the MtZ strain belongs to lineage L4.8 because it has the SNPs in positions 931123 (T/C, *lpqQ*) and 3836739 (G/A, *groEL1*). In total, the MtZ strain has 194 SNPs compared with the H37Rv genome. Twenty-three of them were also present in other L4.8 strains we had sequenced, so we considered that 171 SNPs were specific for the MtZ outbreak strain (**Table S1**).

According to Forrellad et al. and Ramage et al. (Ramage et al., 2009; Forrellad et al., 2013), MtZ isolates had eight non-synonymous SNPs in genes considered as potential virulence factors: seven were specific for the outbreak strain and one was also present in the other L4.8 strains studied (**Table 1**). The ones specific for the strain were in *mce3D* and *mce3F* genes, encoding proteins of the Mce3 cluster, which was shown to be important for virulence in a murine model (Senaratne et al., 2008), but which at the same time is absent from all virulent *Mycobacterium bovis* strains due to the RD7 deletion (Brosch et al., 2002); the *Rv2601A* gene (antitoxin VapB41); the *pks15* gene (two SNPs), whereby the *pks15* gene is likely already a pseudogene in L4 strains due to the frameshift mutation that derives *pks15* and *pks1*, which are corresponding to a functional gene involved in phenolphthiocerol synthesis in L2 strains (Constant et al., 2002); the *virS* gene, part of a virulence operon which controls phagosome–lysosome fusion, acid stress response inside the macrophages, and the expression of enzymes, cell wall and envelope proteins, efflux pumps, ionic transporters, and transcriptional regulators (Singh et al., 2019); and the *Rv3749c* gene (toxin VapC50). The SNP also present in other L4.8 strains was in the *ptpA* gene, involved in host–pathogen interaction and interfering with vesicular trafficking in the macrophage.

**Table 1:** Mutations in genes related to virulence and transmission.

Nucleotide locus	Reference	Variant	Amino acid change	Gene	Functional category	Gene product
2213395	A	G	I181V	*Rv1969*	Virulence, detoxification, adaptation	Mce-family protein Mce3D
2216011–2216012	TG	T	Reading frame alteration	*Rv1971*	Virulence, detoxification, adaptation	Mce-family protein Mce3F
2930254	G	A	G62D	*Rv2601A*	Virulence, detoxification, adaptation	Antitoxin VapB41
3296843	A	G	R290L	*Rv2947c*	Lipid metabolism	Polyketide synthase Pks15
3296972	C	A	V333A	*Rv2947c*	Lipid metabolism	Polyketide synthase Pks15
3447666	T	C	E254G	*Rv3082c*	Virulence, detoxification, adaptation	Virulence-regulating transcriptional regulator VirS
4197895	C	G	K81N	*Rv3749c*	Conserved hypotheticals	Toxin VapC50
2507254*	G	A	A37T	*Rv2234*	Regulatory proteins	Phosphotyrosine protein phosphatase PtpA
24445	C	G	V334L	*Rv0020c*	Regulatory proteins	Conserved protein with FHA domain, FhaA
113371	A	G	H1124R	*Rv0101*	Lipid metabolism	Peptide synthetase Nrp
1460992	A	C	H250P	*Rv1304*	Intermediary metabolism and respiration	ATP synthase a chain AtpB
219104-219105	GA	G	Reading frame alteration	*Rv0187*	Intermediary metabolism and respiration	O-methyltransferase
3017134*	G	T	A93S	*Rv2702*	Intermediary metabolism and respiration	Polyphosphate glucokinase PpgK
4144477	G	A	T147I	*Rv3701c*	Conserved hypotheticals	Conserved hypothetical protein
226475	C	T	A33T	*Rv0193c*	Conserved hypotheticals	Hypothetical protein
1273071*	T	C	S217P	*Rv1145*	Cell wall and cell processes	Conserved transmembrane transport protein MmpL13a
1504420	G	A	R22H	*Rv1337*	Cell wall and cell processes	Integral membrane protein
2094479	T	C	D237G	*Rv1844c*	Intermediary metabolism and respiration	6-Phosphogluconate dehydrogenase Gnd1
2373539	C	T	P304S	*Rv2113*	Cell wall and cell processes	Integral membrane protein
2373696	A	ACCG	Reading frame alteration	*Rv2113*	Cell wall and cell processes	Integral membrane protein
2709030	G	T	F312L	*Rv2411c*	Conserved hypotheticals	Conserved hypothetical protein
3610610	G	A	Q194 STOP	*Rv3234c*	Lipid metabolism	Triacylglycerol synthase Tgs3

Following the functional classification used in Mycobrowser, the SNPs in genes considered to be virulence factors are colored in orange. In green are genes classified as “required for survival in primary murine macrophages” and “required for growth in C57BL/6J mouse spleen.” In blue are genes classified as “disruption of this gene provides a growth advantage for in vitro growth”.

*Present also in other L4.8 strains. All the points refer to the H37Rv genome.

We also checked the non-synonymous SNPs in genes that Mycobrowser classified in some of these categories: “required for survival in primary murine macrophages” (Rengarajan et al., 2005), “required for growth in C57BL/6J mouse spleen” (Sassetti et al., 2003), and “disruption of this gene provides a growth advantage for *in vitro* growth” (DeJesus et al., 2017). The first two are related to infection ability, so we grouped both. MtZ has SNPs in *Rv0020c*, *Rv0101*, *Rv0187*, *Rv0193c*, *Rv1145*, *Rv1304*, *Rv1337*, *Rv1844c*, *Rv2113*, *Rv2113*, *Rv2411c*, *Rv2702*, *Rv3234c*, and *Rv3701c.* Information about these genes can be found in **Table 1**.

Any of the sequenced isolates had SNPs in genes related to first-line drug resistance; however, case 124 was the only one showing molecular resistance to quinolones (*gyrA*, D94G). Among the non-sequenced isolates, case 31 showed phenotypic resistance to rifampicin, case 174 to ethambutol, case 181 to isoniazid and ethambutol, and case 239 to isoniazid. The rest of the isolates were susceptible to all antituberculosis drugs.

### Epidemiological Study

**Figure 1:**
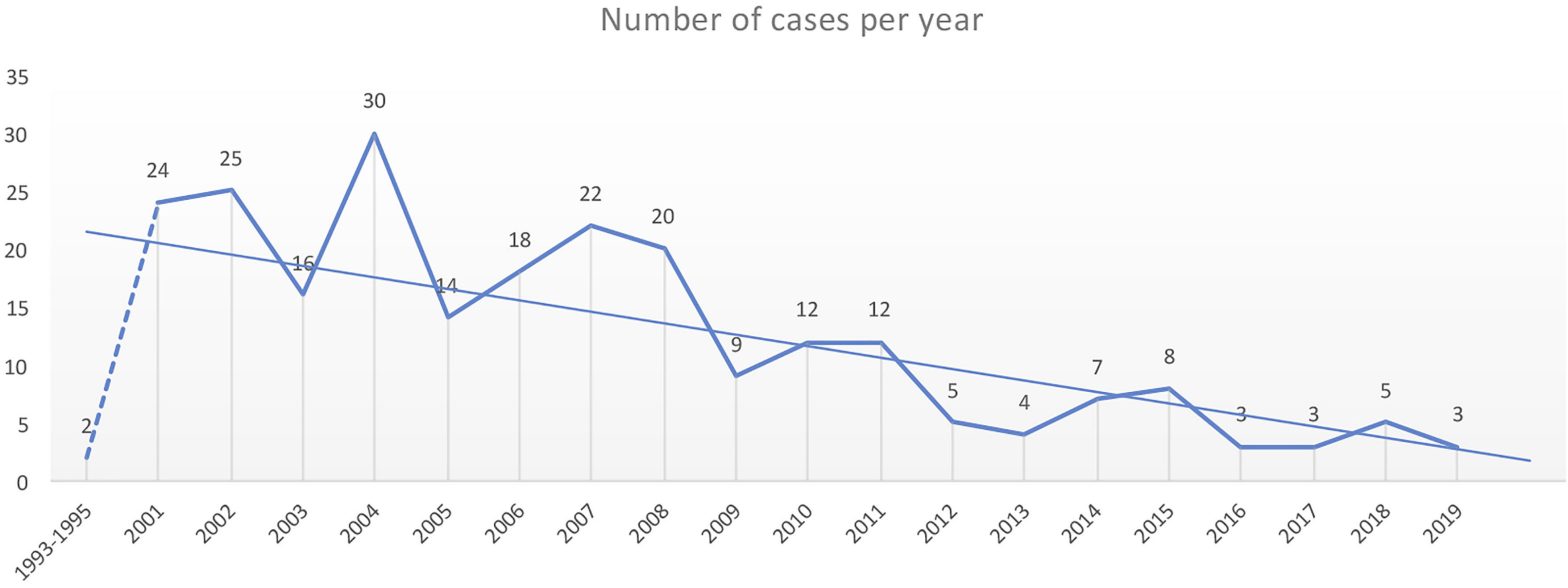
Number of cases of the *Mycobacterium tuberculosis Zaragoza* (MtZ) strain during the study period. The two cases in 1993–1995 were identified after the discovery of the strain in the 2001–2004 study. The highest number of cases was in 2004, with 30 cases, and the number continued to be high during the first decade of the 2000s. The number of cases diminished in 2009, and in the last few years, it has been quite low. No cases were diagnosed in 2020.

Until December 2020, 242 cases of MtZ distributed throughout the region were detected. The number of cases per year can be found in **Figure 1**. The highest number of cases corresponded to the first decade of the 2000s, with a special rise in 2004. In 2009, there was a drop in the number of cases, which has continued to date. Only two cases of MtZ were identified for 1993–1995, both presenting an extrapulmonary form of the disease.

Focusing on the epidemiological characteristics of the population affected by this strain, the more affected group of age was between 31 and 45 years (92 cases), followed by the 16–30 group (66 cases). The majority of the cases were autochthonous, with only 27 being foreign-born (coming from 17 different countries). Sixty percent of cases had a positive smear result (BK+), and 53 cases had an X-ray result as cavitated pathology and 80 as non-cavitated pathology. More detailed information can be found in **Table 2**.

**Table 2:** Characteristics of the 242 TB cases involved in the MtZ outbreak.

	*N* (%)
**Age (years)**	
0–15	17 (7.0)
16–30	66 (27.3)
31–45	92 (38.0)
46–60	49 (20.2)
>60	18 (7.4)
**Intravenous drug users**	
Yes	17 (7.0)
No	124 (51.2)
Unknown	101 (41.8)
**Smokers**	
Yes	92 (38.0)
No	40 (16.5)
Unknown	110 (45.5)
**HIV status**	
Positive	24 (9.9)
Negative	152 (62.8)
Unknown	66 (27.3)
**Alcohol consumption**	
High	43 (17.8)
Moderate	11 (4.5)
Low/no	79 (32.7)
Unknown	109 (45.0)
**X-ray result**	
Normal	2 (0.8)
Cavitated pathology	53 (21.9)
Non-cavitated pathology	80 (33.1)
Unknown	107 (44.2)
**Bacilloscopic (BK) result**	
Positive	145 (59.9)
Negative	90 (37.2)
Unknown	7 (2.9)

**Figure 2:**
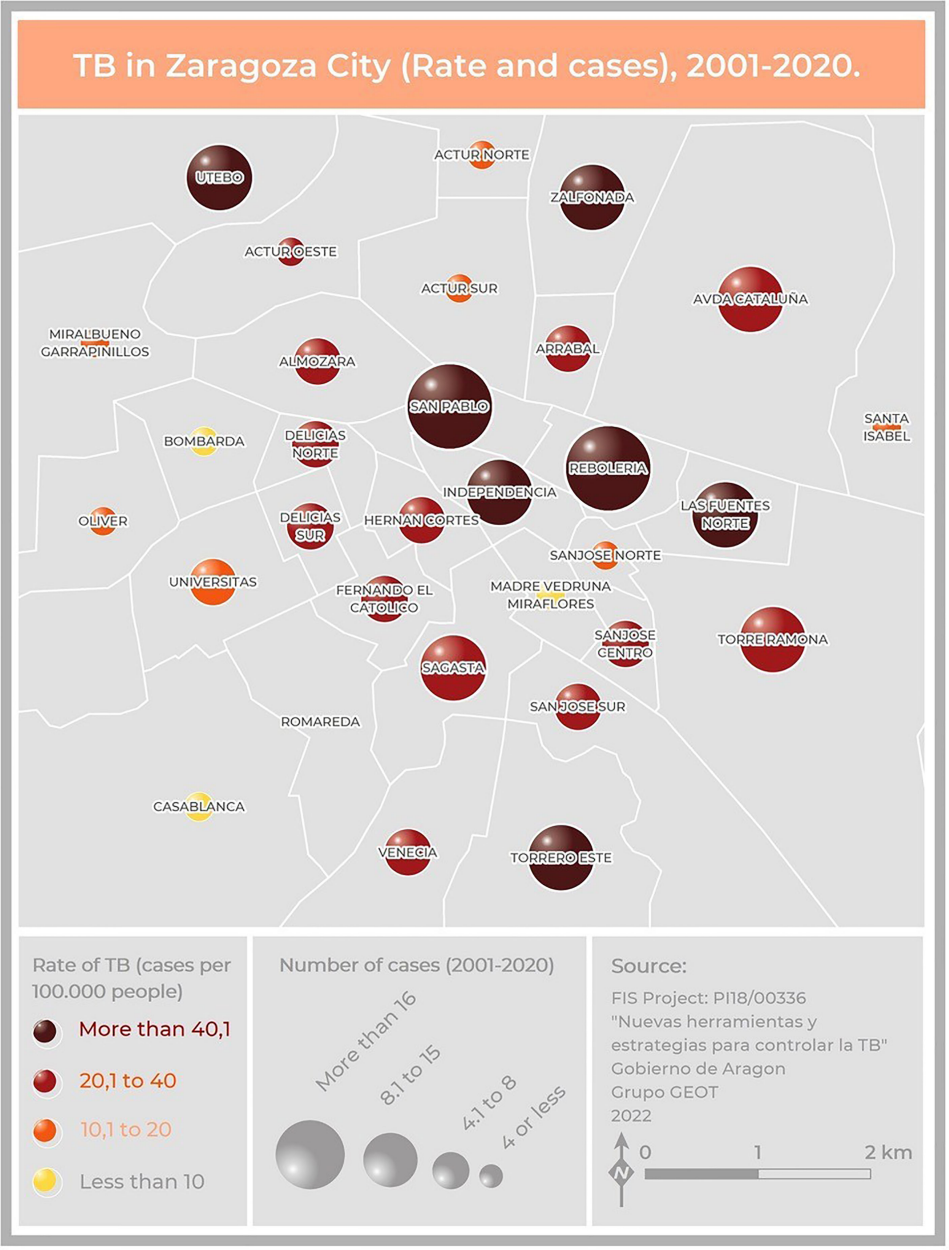
Map of Zaragoza City showing the number of cases and the incidence of TB due to the MtZ strain in the different neighborhoods. The map represents two variables: TB incidence per 100,000 population, represented by color hue and lightness (yellow to brown), and number of cases, represented by size. San Pablo and Rebolería, in the old town, are the neighborhoods with a higher incidence and number of cases. Cases detected outside the city were not included.

Two hundred and five cases lived in Zaragoza City, the community capital. The rest of the cases lived in smaller cities or villages. The cases were distributed among 40 health zones, and the map related to the TB rate in Zaragoza showed that the highest concentrations were in the city center (San Pablo, Independencia, Rebolería) and the outer edges (Utebo, Torrero) (**Figure 2**). In general, higher rates corresponded to socioeconomically vulnerable areas (San Pablo, Rebolería, Las Fuentes, Torrero), with one exception (Independencia). There was spatial contiguity between the areas with the highest values and basic spatial patterns can be seen: concentric behavioral patterns (center, intermediate pattern, peripheral pattern). Moreover, a map locating all the cases was constructed in order to study possible links related to neighborhood contacts (data not shown).

Some epidemiological links were found in the previous study of López-Calleja et al. in 2009 (López-Calleja et al., 2009): nine cases (cases 76, 78, 79, 80, 81, 82, 83, 84, and 85) were part of a nursery outbreak, involving eight babies and their carer. Case 3 was the uncle of case 10 and the cousin of case 13. Cases 5 and 57 were father and son, as were cases 40 and 46. Cases 70 and 71 were married, as were cases 16 and 20. Cases 54 and 95 lived in the same building, but no specific link could be established between them. The same occurred for cases 38 and 48. Four cases (cases 6, 32, 45, and 55) were in prison. New epidemiological investigations revealed that cases 115 and 126 were brother and sister, and case 115 was a workmate of case 129. Cases 122 and 140 lived in the same building, and cases 221 and 222 were married.

### Genomic Study

**Figure 3:**
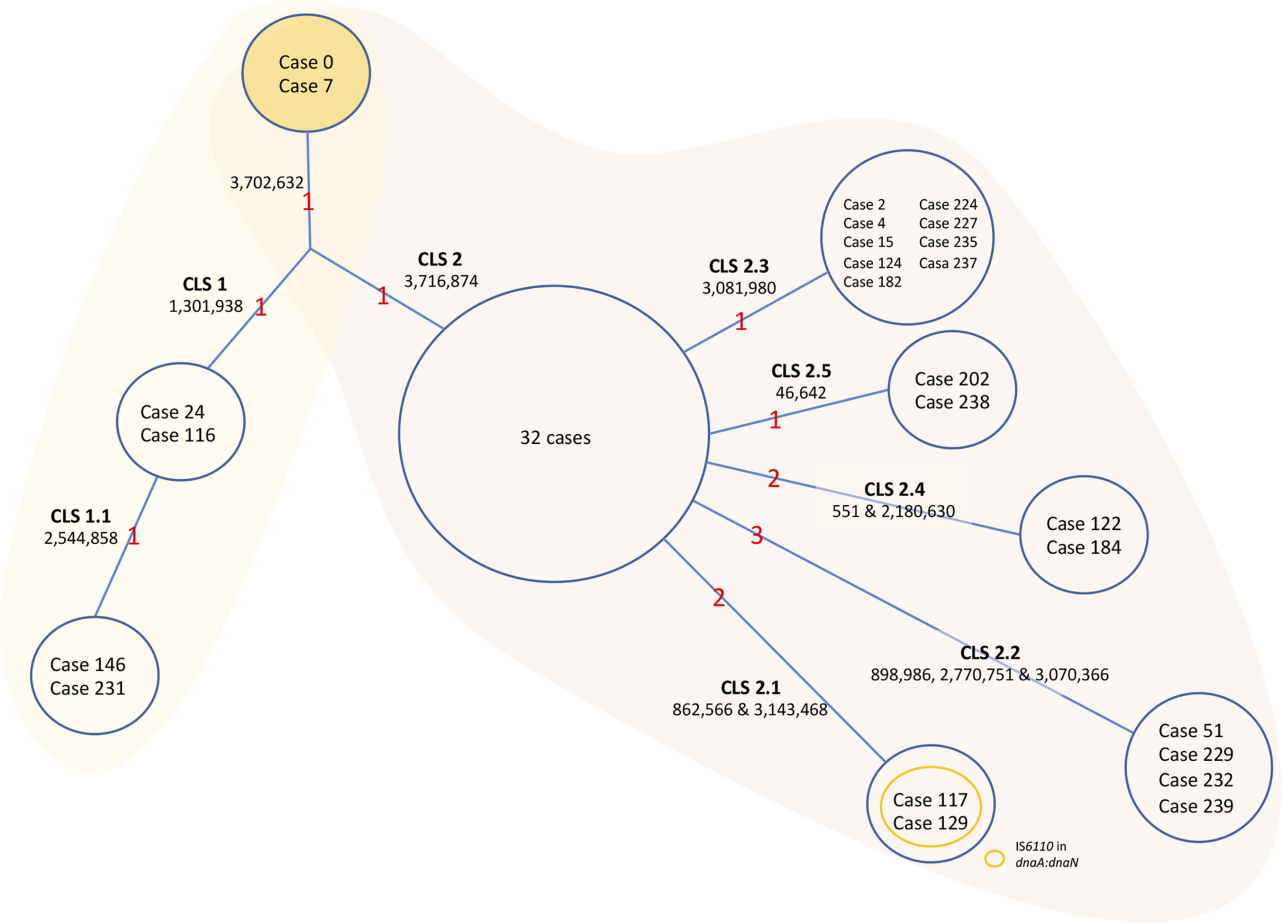
Evolution of the MtZ strain. One acquired SNP at point 3702632 separates Cases 0 and 7, who share the original base, from the rest of the sequenced cases. A Case x must exist, though not sequenced, from which CLS-1 and CLS-2 emerged. CLS-1 emerged after the acquisition of a single nucleotide polymorphism (SNP) at position 1301938. A sub-CLS (CLS-1.1) emerged from this after the acquisition of another SNP at point 2544858. CLS-1 was mostly located in the Huesca area. We could classify cases 65, 113, 139, and 216 in this CLS-1 by PCR. Formerly, an SNP acquired from the hypothetical case x at point 3716874 led to CLS-2, in which the majority of MtZ cases are included. The red numbers are the number of acquired SNPs. The cases appearing in the figure are the ones sequenced. The unique SNPs were excluded from the figure to simplify the data.

The sequence analysis of the genomes was carried out in BioNumerics software. The data are summarized in **Figure 3** for simplicity. Only two isolates could be considered as the original sequence of the strain (case 0, belonging to the period of 1993–1995, and case 7 from 2001). The rest of the isolates had an SNP in position 3702632; therefore, there had to be an intermediate isolate that gained this mutation that was not sequenced (case x). From this intermediate isolate, two branches appeared. Several cases conserved the original base at position 3716874 (CLS-1), while the rest of the isolates had an SNP in that position (CLS-2) (**Figure 3**).

In addition, isolates from CLS-1 also shared an SNP at position 1301938, and two of the isolates shared one more SNP at position 2544858, indicating transmission between these two cases (CLS-1.1). There were no epidemiological links among cases of CLS-1, except that three of them lived in the area of Huesca, one of the provincial capitals. A few more cases, not sequenced, also lived in this area. We confirmed by PCR that four of these cases had the characteristic SNPs of CLS-1. One sequenced case who lived in this area was classified in CLS-2, therefore not sharing these SNPs.

**Figure 4:**
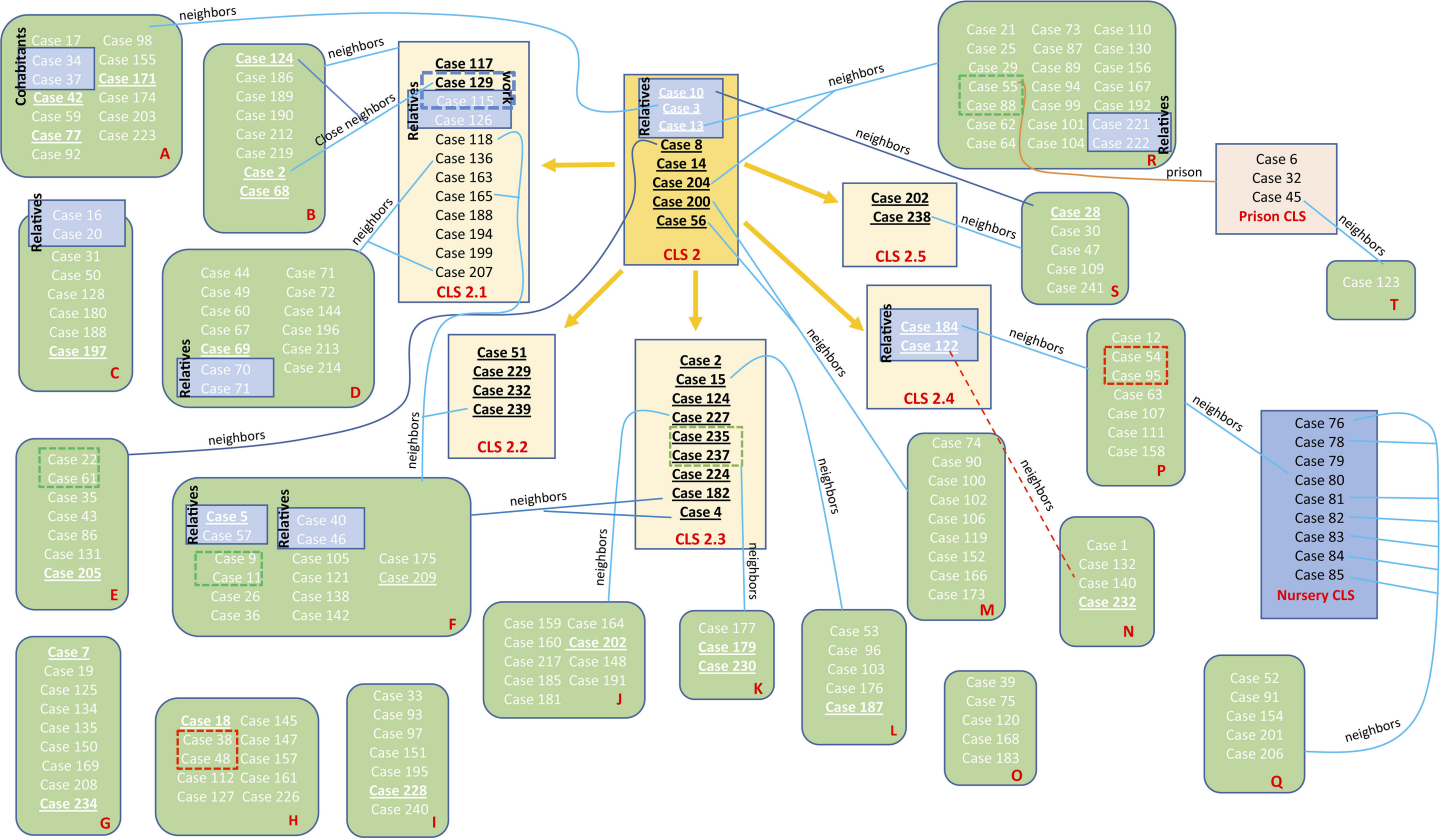
Schematic view of the transmission chain for CLS-2 with regard to the molecular and epidemiological links. Five transmission CLSs emerged from CLS-2, the original sequence of this MtZ branch. Epidemiological links are included in the picture. Each red letter corresponds to a different neighborhood. Those cases presented in a blue box had a confirmed epidemiological link (relatives, work contact, and the nursery cluster). Those in a red dotted box or line live at the same building although no familiar link was established (probable epidemiological link). Those cases in a green dotted box are nearby neighbors (living on the same street). Those underlined are the ones sequenced. Cases 2, 124, 188, 202, and 232 appear twice (in the sub-CLS and the neighborhood they belong to) in order to simplify the diagram.

Eight cases of CLS-2 were considered the original sequence of this cluster, as they only shared the SNPs in positions 3702632 and 3716874. The majority of these cases were diagnosed in 2001, at the beginning of the outbreak. Twenty-four more cases shared those SNPs, having some other unique SNPs, not transmitted to other isolates; therefore, 32 isolates could be considered as part of the CLS-2 original sequence. From CLS-2, five more subclusters appeared. These isolates conserved the two characteristic SNPs of CLS-2 together with independent SNPs, resulting in five new transmission sub-CLSs. The genetic and epidemiological links among cases of CLS-2 can be found in **Figure 4**.

Isolates of CLS-2.1 shared the SNPs at positions 862566 and 3143468. In addition, they had an extra IS*6110* inserted in *dnaA:dnaN*. Only two cases among the sequenced isolates had those SNPs. However, 10 more cases could be included in this cluster after the study of IS*6110* in *dnaA:dnaN*. Moreover, the majority of CLS-2.1 cases lived in the same neighborhood.

Isolates of CLS-2.2 shared three SNPs at positions 898986, 2770751, and 3070366. Four sequenced isolates belonged to this cluster. Nine cases belonged to CLS-2.3, sharing an SNP at position 3081980, and two isolates constituted CLS-2.4 sharing the SNPs at positions 551 and 2180630. Finally, CLS-2.5 was formed by two isolates that shared the SNP at position 46642. Additional information about these transmitted SNPs in the different subclusters can be found in **Table 3**.

**Table 3:** SNPs transmitted in the different CLSs of the MtZ outbreak.

Point	Gene	Amino acid change	Mutation effect	Functional category	Gene description	CLS
**551**	*dnaA*	R184Q	Non-synonymous (deleterious)	Information pathways	Plays an important role in the initiation and regulation of chromosomal replication.	CLS-2.4
**46642**	*Rv0042c*	L189R	Non-synonymous (deleterious)	Regulatory proteins	Possibly involved in transcriptional mechanism	CLS-2.5
**862566**	*Rv0769*	G52D	Non-synonymous (deleterious)	Intermediary metabolism and respiration	Dehydrogenase/reductase	CLS-2.1
**898986**	*Rv0805*	–	Synonymous	Intermediary metabolism and respiration	Hydrolyzes cyclic nucleotide monophosphate to nucleotide monophosphate	CLS-2.2
**1301938**	*PE12*	–	Synonymous	Pe/ppe	PE family protein PE12	CLS-1
**2180630**	*Rv1928c*	I196M	Non-synonymous (neutral)	Intermediary metabolism and respiration	Short-chain type dehydrogenase/reductase	CLS-2.4
**2544858**	*lppN*	A54V	Non-synonymous (neutral)	Cell wall and cell processes	Lipoprotein LppN	CLS-1.1
**2770751**	*pepN*	V589A	Non-synonymous (deleterious)	Intermediary metabolism and respiration	Aminopeptidase with broad substrate specificity to several peptides	CLS-2.2
**3070366**	*Rv2757c*	W74L	Non-synonymous (deleterious)	Virulence, detoxification, adaptation	VapC21 toxin	CLS-2.2
**3081980**	*dapB*	R121Q	Non-synonymous (neutral)	Intermediary metabolism and respiration	Involved in the biosynthesis of diaminopimelate and lysine from aspartate semialdehyde	CLS-2.3
**3143468**	*dinF*	L54P	Non-synonymous (deleterious)	Information pathways	Induction by DNA damage	CLS-2.1
**3702632**	*deoA*	L279R	Non-synonymous (deleterious)	Intermediary metabolism and respiration	Catalyzes the reversible phosphorolysis of pyrimidine nucleosides	CLS-1 and CLS-2 (including the subclusters)
**3716874**	*dacB1*	–	Synonymous	Cell wall and cell processes	Involved in peptidoglycan synthesis	CLS-2 (including the subclusters)

Points in the genome and the affected genes are in reference to the H37Rv strain. The amino acid change, the kind of mutation and its potential effect (according to PROVEAN), the functional category (according to Mycobrowser), and information concerning the genes are supplied.

A strain was considered to belong to a cluster when there were ≤12 SNPs between at least two isolates of the potential outbreak. If there were ≤5, it was considered recent contact (Lalor et al., 2018). A table with the genomic distance among the different isolates of the MtZ outbreak was constructed (**Table S2**). All the isolates had ≤12 SNPs with at least one isolate, meaning all of them belonged to the MtZ outbreak, and 50 isolates had recent contact (≤5 SNPs) with at least one isolate.

We have followed the classification established by Lalor et al. in 2018 for the different links among the outbreak cases (Lalor et al., 2018). For the MtZ outbreak, we found several “confirmed epidemiological links” as relatives, husband and wife, cohabitants, work contact, and the nursery cluster. “Probable epidemiological links” could be established among the cases in prison and cases who live in the same building, as they spent time in the same location, but the timing was unknown. Finally, possible epidemiological links could be established among cases who lived in the same area. These detailed links can be found in **Figure 4**.

**Figure 5:**
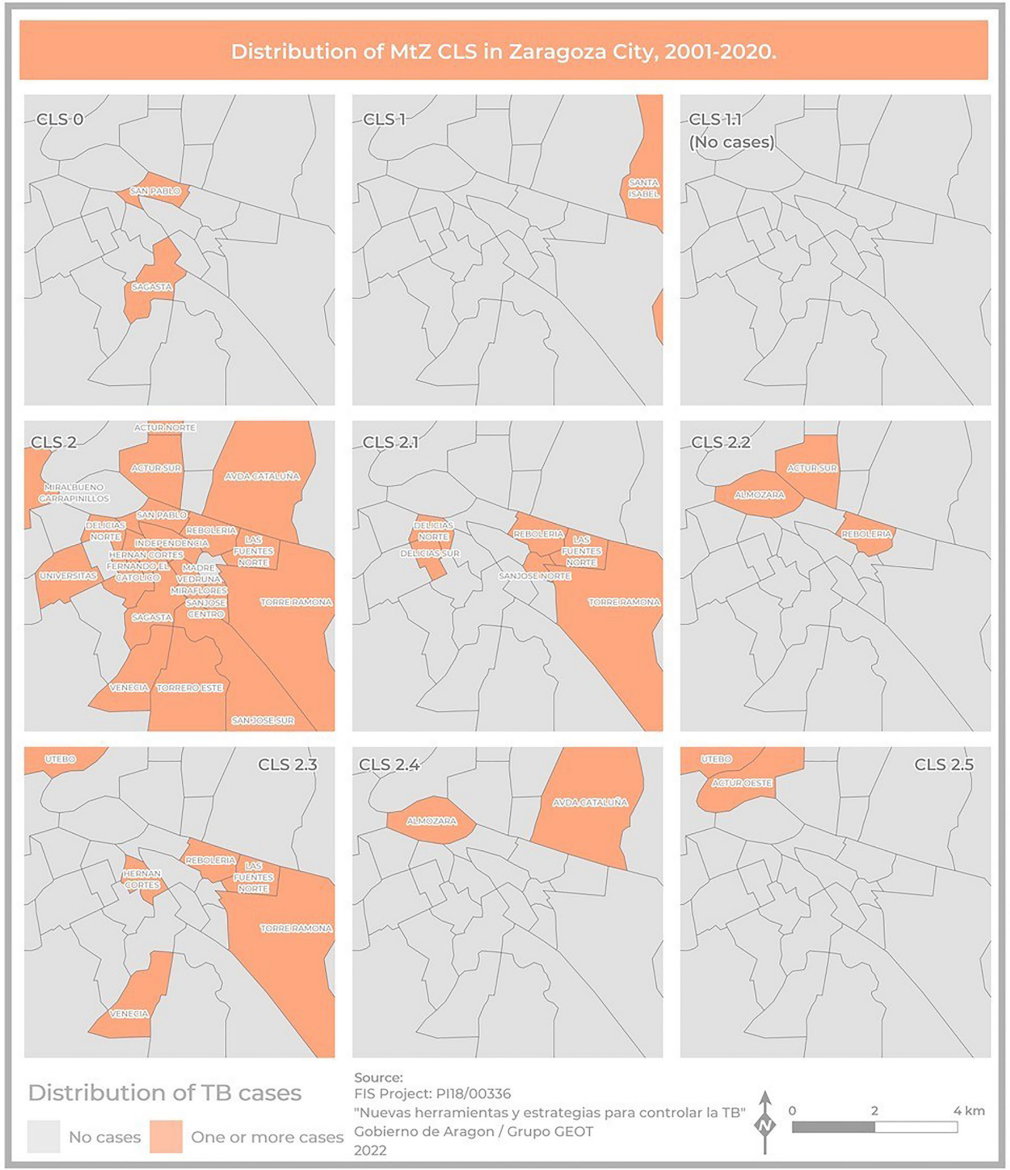
Map showing the geographical distribution of MtZ CLSs within Zaragoza City, using a presence–absence legend (orange–gray). CLS-2 was the most widespread, involving the highest number of neighborhoods. There were no cases of CLS-1.1 because they were in the Huesca area, another province of the community.

The geographical distribution of the different MtZ CLSs in Zaragoza City is shown in **Figure 5**. The TB case distribution map reveals an uneven distribution by CLS. The CLS with the highest presence in the city was CLS-2 (17 areas), followed by CLS-2.1 (six areas) and CLS-2.3 (six areas). In some CLSs, there were no cases in the city at all (CLS-1.1).

### RNAseq and PE_PGRS Secretion Studies

**Figure 6:**
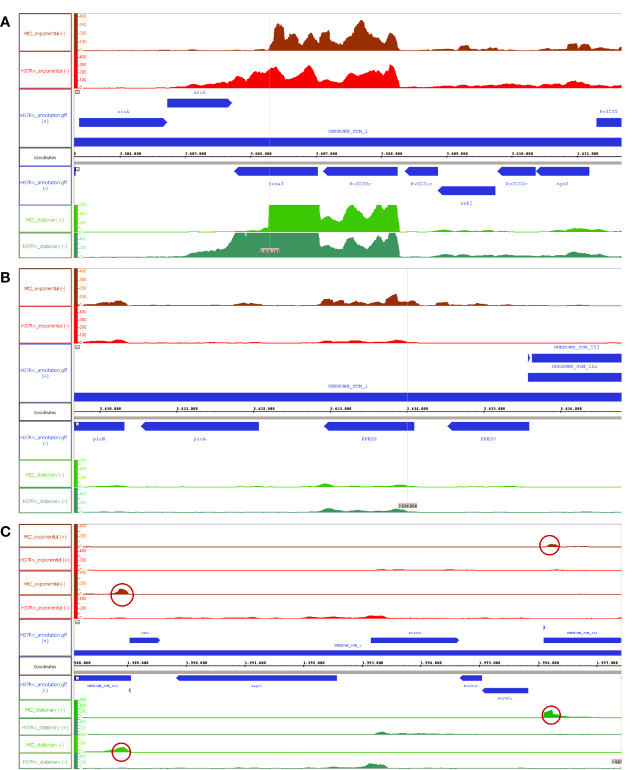
Effects of the IS*6110* in the transcription of the MtZ strain. **(A)** Transcriptomic profile of MtZ and H37Rv in the *desA3* gene region. The transcription is interrupted in MtZ. **(B)** Transcriptomic profile of MtZ and H37Rv in the *ppe38* gene region. This gene is overexpressed in MtZ in the exponential growth phase. **(C)** Transcriptomic profile of the *cut1:Rv1765c* region. The absence of transcription confirms the loss of the intermediary genes. The peaks circled in red coincided with IS*6110* copies in the genome of the H37Rv strain showing overexpression in MtZ. For all pictures: above is the exponential growth phase (in red colors) and below is the stationary growth phase (in green colors). In the center is the annotation for the H37Rv reference strain (in blue color). “−” is the negative DNA strand; “+” is the positive DNA strand.

In order to analyze the effect of IS*6110* in the adjacent genes of the genome of the MtZ strain, we performed a transcriptomic study using three different MtZ isolates: case 0, considered the original isolate with 12 IS*6110* copies; case 129, which belonged to a CLS-2.1 isolate with an extra IS*6110* in *dnaA:dnaN*; and case 241, which lost one IS*6110* copy due to recombination between two of them around the DR region. The study was carried out in both exponential and stationary growth phases. Relevant findings can be found in **Figure 6**.

The transcriptional analysis allowed us to observe three different effects of IS*6110* for the three MtZ isolates in both growth phases. First, transcription was interrupted in the *desA3* gene upon reaching the IS*6110* insertion site (3606310) while continuing in the H37Rv reference strain (**Figure 6A**). Second, overexpression of the *ppe38* gene in the exponential growth phase, where the MtZ strain had an IS*6110* inserted at point 2633977, was observed (**Figure 6B**). Finally, the transcriptomic study confirmed the lack of transcription (zero reads) of all the genes lost in the region *cut1:Rv1765c* due to the recombination of two close IS*6110* (**Figure 6C**).

Focusing on the different IS*6110* copies among the three isolates analyzed, nothing different was observed for the *dnaA:dnaN* region in case 129 compared with the reference strain or with the other two MtZ isolates. For the case 241 isolate, the adjacent genes to the DR region had no transcription, confirming the deletion of genes *Rv2816c* to *Rv2823c.*

Analyzing the transcriptional areas of IS*6110* using H37Rv locations, we observed higher transcription peaks for the MtZ isolates compared with the H37Rv reference strain, being even higher in the stationary phase, thus meaning that IS*6110* was overexpressed in the MtZ isolates (**Figure 6C**). No major differences were found among the transcriptomes of the three MtZ isolates studied.

**Figure 7:**
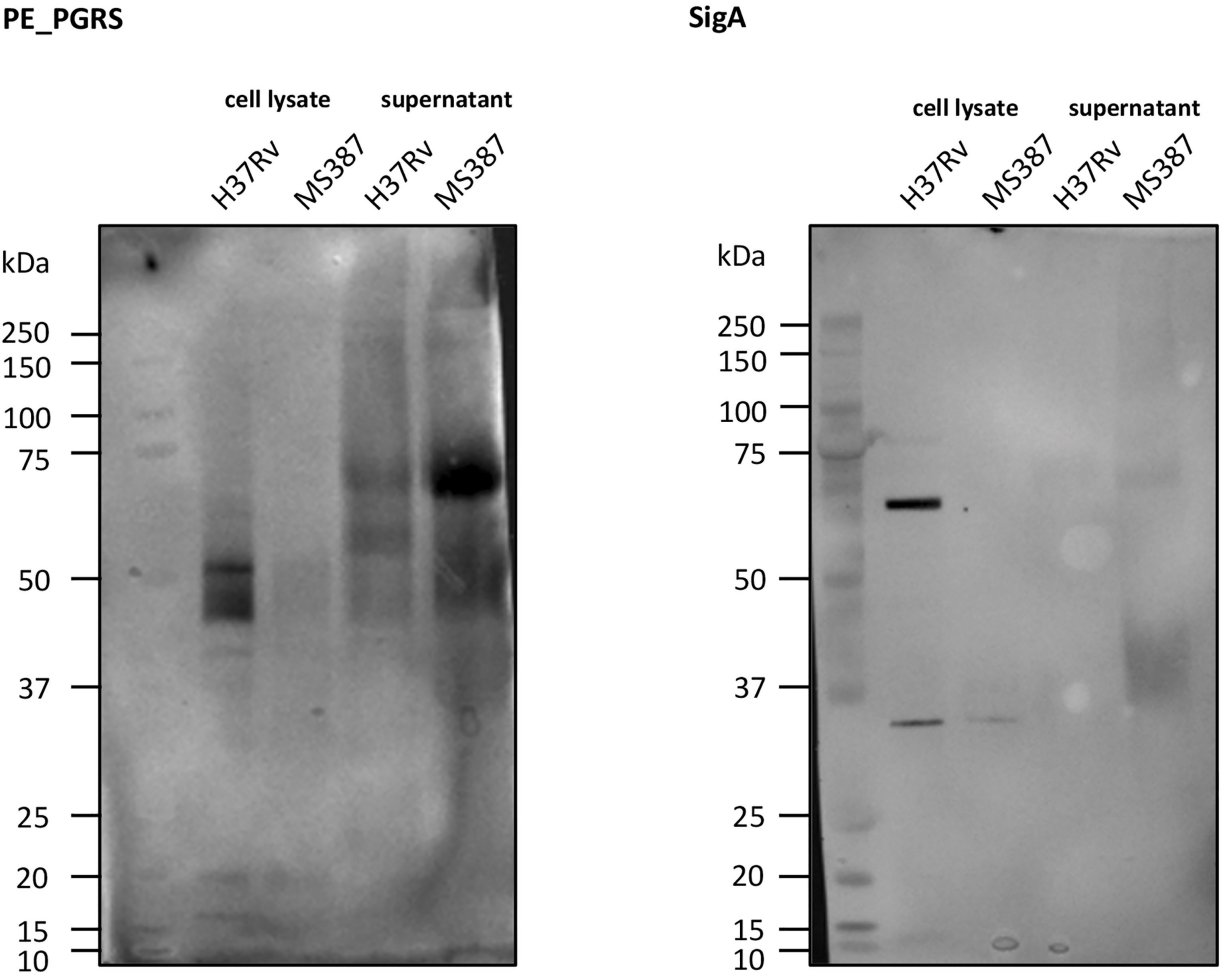
Western blot of PE_PGRS secretion. As can be observed, MS 387 (MtZ strain, case 0) secretes PE_PGRS, even in a higher amount than the reference H37Rv strain. sigA control did not appear in MS 387 because the protein concentration in the cell lysate was too low.

It has been observed (Ates et al., 2018) that MTB strains belonging to the Beijing lineage, often thought to be hypervirulent, are defective in the secretion of PE_PGRS (polymorphic GC-rich repetitive sequences) proteins and that the secretion of more than 80 proteins of this protein family is dependent on the presence of at least one copy of *ppe38*/*ppe71* genes. Ates et al. (2018) demonstrated a possible link between the hypervirulent phenotype of some Beijing strains and changes in this genomic region caused by IS*6110* insertion and/or recombination between *ppe38/71*. As MtZ also has both of these genes targeted by IS*6110* insertions, we wanted to investigate whether PE_PGRS secretion was affected in this strain. Interestingly, the analysis of the PE_PGRS secretion by Western blot revealed that the MtZ strain produced and secreted PE_PGRS, even in higher amounts than H37Rv, used as reference (**Figure 7**), suggesting that the specific *IS6110* insertions did not disrupt the functionality of the remaining PPE proteins.

## Discussion

The MtZ strain is the most successful strain ever documented in the Aragon region, circulating from the 1990s until now. Thanks to the molecular surveillance protocol of TB carried out in Aragon, all MTB clinical isolates are genotyped (except for those during 1996–2000). At the same time that MtZ arose, something similar was happening in other parts of the world: strain GC in Gran Canaria (Caminero et al., 2001), strain C in New York (Friedman et al., 1997), strain CH in Leicester (Watson and Moss, 2001), the Harlingen strain in the Netherlands (van Soolingen et al., 1999), and the Danish cluster 1 and 2 strains in Denmark (Lillebaek et al., 2004). All these strains, MtZ included, had in common high transmissibility and drug susceptibility. It is difficult to understand how a drug-susceptible strain of *M. tuberculosis* can spread so widely among the first-world population. A higher capacity of infection could explain how this strain became responsible for such a large proportion of TB cases in a city with a strong health coverage. On the other hand, the cases were correctly treated, and consequently, few drug resistances developed. The observed tendency suggests that the outbreak is about to conclude, as no cases were diagnosed in 2020; however, at least one case was detected in 2021 (not included in the study).

The development of WGS has allowed a deeper study of the molecular characteristics of outbreak strains. MtZ belongs to L4.8, the closest phylogenetic branch to the reference strain H37Rv (L4.9), showing less than 200 SNPs between them We could confirm the molecular susceptibility to all the first-line drugs in all cases; however, molecular monoresistance to quinolones was found in case 124, a great advantage of WGS over other genotyping methods, offering a global vision of the genetic resistance pattern of the isolates. This resistance could have developed as a consequence of a previous treatment since a residual previous TB was observed in the patient’s thorax X-ray.

Moreover, the MtZ strain had eight SNPs in genes that have been listed as virulence factors (Ramage et al., 2009; Forrellad et al., 2013) but whose real impact on virulence remains to be demonstrated. Additionally, mutations in 14 genes that could be somehow related to *in-vivo* growth, pathogenesis, and survival in the murine host (Sassetti et al., 2003; Rengarajan et al., 2005; DeJesus et al., 2017) could also interfere with the specific dominance of this strain. Regarding the SNPs in potential virulence factors, *mce* genes have been traditionally related to cell entry (Arruda et al., 1993). For the *mce3* operon, this function was demonstrated for the *mce3C* gene (Zhang et al., 2018); however, the complete *mce3* operon seems more related to lipid transport (Klepp et al., 2022). The MtZ strain has two SNPs in this operon, one of them a frameshift, suggesting a possible defect in this lipid transport. Nevertheless, it has been shown that the different *mce* operons can interact with each other (Klepp et al., 2022); therefore, complementation between systems could be possible. In addition, the entire *mce3* operon is absent from *M. bovis* and most other animal-adapted strains of the *M. tuberculosis complex*, suggesting that it is not required for infecting the macrophages and preventing that the frameshift SNP attenuates the MtZ strain. Concerning the two SNPs in the *pks15* gene, they might affect the lipid metabolism, but this remains unclear, as *pks15* is already a pseudogene in L4 strains (Constant et al., 2002). Some SNPs also suggest that MtZ could have a better survival ability inside the macrophages: the *virS* gene which controls the vesicular trafficking and survival inside the macrophages regulating the host immune system (Singh et al., 2019); and the *ptpA* gene, responsible for the inhibition of macrophage phagosome–lysosome fusion and phagosome acidification (Bach et al., 2008; Wong et al., 2011; Poirier et al., 2014; Wang et al., 2015; Wang et al., 2016). The toxin–antitoxin systems play an important role in stress response and genome stability (Ramage et al., 2009), so the two SNPs found in *VapB41* and *VapC50* genes could also be affecting the success of the infection. Considering all this, some of these mutations could be related to the high transmissibility and success of the MtZ strain.

The explosion of cases took place at the beginning of the 2000s, as only two cases were detected for 1993–1995 (with no data for 1996–2000). The six first cases affected with the MtZ strain in 2001 were diagnosed over the course of a few days, and the outbreak turned into the largest one ever experienced in the region. In a study carried out by Hamblion et al. in 2016 (Hamblion et al., 2016) regarding clustering in London for a 3-year period, they observed that if the two first cases of an outbreak are diagnosed with less than 90 days between them, it is probable that the outbreak becomes larger. Genomic information revealed the gain of two mutations (points 3702632 and either 3716874 or 1301938) for almost all MtZ cases since 2001; therefore, both facts may be related to its successful spread in the population. However, none of those SNPs is in a gene considered to be a virulence factor or relevant for pathogenesis.

It was curious that people infected with the MtZ strain did not have, in general, any risk factor, which was already observed by López-Calleja et al. (2009) for the first 85 cases. Different from an X-family outbreak which also began in the 1990s among the local population and with a high proportion of HIV^+^ individuals, users of intravenous drugs, and prisoners (Comín et al., 2021), the MtZ outbreak did not show a high percentage of these individuals, just a high proportion of smokers (38%) and a moderate proportion of alcohol dependence (18%). On the other hand, in both outbreaks, young people were the most affected group, with more than 70% of the cases under 45 years old.

Although some links were established using epidemiological information, the genomic study revealed additional transmission chains, as well as the evolution of the strain. From cases 0 and 7, an SNP was gained at position 3702632 (case x, missing from our study). Subsequently, one SNP in 1301938 led to CLS-1, and another in 3716874 led to CLS-2. CLS-2 comprised the largest number of isolates studied, and five subclusters arose from it. As a result of the WGS analysis, a higher number of cases could be included in the transmission chain than just those connected by epidemiological links. However, the direction of the transmission could not be established in the majority of cases, something recurrent in TB outbreak studies (Casali et al., 2016; Hatherell et al., 2016; Lalor et al., 2018; Comin et al., 2020; Comín et al., 2021). As an example, cases 51 and 229 from CLS-2.2 had an identical genomic sequence, but case 51 was diagnosed 15 years before, so we think that case 51 would be the first case, as transmission always moves toward SNP accumulation (Schürch et al., 2010). Case 51 and/or case 229 or other related but not sequenced isolates should have infected case 232 and case 239 (also belonging to CLS-2.2), which accumulated more SNPs. Another extra difficulty to study the TB outbreaks and the reconstruction of the transmission chain is the latency period, as the infection could take place years before the diagnosis (Salgame et al., 2015). We observed this phenomenon, for example, for cases 5 and 57 (WGS not available), who were father and son, respectively. The father was diagnosed in 2001, probably when he infected his son, who developed the disease 2 years later, in 2003.

Regarding the SNPs transmitted among the different clusters, four of them caught our attention for being in genes related to virulence and pathogenesis. An SNP in the *Rv0805* gene, which regulates the intracellular concentration of cAMP and could have been altering the properties of the cell wall, was transmitted among cases of CLS-2.2 (Shenoy et al., 2005; Podobnik et al., 2009). In addition, an SNP in the *pepN* gene, which encodes for an M1 family zinc metallo-aminopeptidase that plays a crucial role in survival, cell maintenance, growth and development, virulence, and pathogenesis, was also transmitted among CLS-2.2 isolates (Ingmer and Brøndsted, 2009). It has been hypothesized that PepN may cleave pathogen and host proteins in host macrophages to regulate virulence levels (Sharma et al., 2019). A third SNP transmitted in CLS-2.2 was in the *Rv2757c* gene, VapC21 toxin, whose importance in pathogenesis has been described previously as part of a toxin–antitoxin system (Ramage et al., 2009). We could not find any isolate with only one or two of these SNPs, as if they occurred in a single isolate that later was transmitted. Finally, an SNP transmitted among cases of CLS-2.3 was in the *dapB* gene, an essential reductase whose inhibition blocks the production of meso-diaminopimelate, leading to inhibition of *de-novo* lysine biosynthesis and peptidoglycan assembly. Both of these pathways are crucial for the survival of the pathogen (Usha et al., 2012). Among the subclusters obtained by WGS, CLS-2.2 and CLS-2.3 were the largest, which may be related to the presence of some of these SNPs. Another important fact is the extra IS*6110* inserted in *dnaA:dnaN* observed for CLS-2.1. This variant was transmitted to 12 isolates over the entire period considered by the study, so the variant persisted in the population. We do not know if this copy conferred any biological advantage, but at least it did not seem to be detrimental to the bacteria. Nothing remarkable was found in the transcriptomic study related to this additional IS*6110* copy for this MtZ variant.

Regarding the genetic distances, the majority of isolates could be considered recent contact (≤5 SNPs) with at least one isolate. Six cases were more distant (≥5 and ≤12), but it is important to have in mind that the strain was circulating for many years, so evolutionary SNPs occurred. In addition, many unique SNPs detected in certain isolates could either have been generated in the culture process or have been introduced by the sequencing.

In order to better understand the role of the multiple IS*6110* copies in the genome, a transcriptomic study was carried out in three MtZ isolates that differed for one IS*6110* copy. As we knew the location of all the copies, we wondered about the role of this element in the transcription of the adjacent genes. This analysis allowed us to confirm different pieces of evidence. First, we could see the three general effects that the insertion of the IS*6110* could produce in the genome: the disruption of the gene in which it has been inserted (*desA3*) (Sampson et al., 1999; Beggs et al., 2000; Warren et al., 2000; Yesilkaya et al., 2005), the deletion or inversion of the DNA in between (*cut1:Rv1765c*) due to recombination (Sampson et al., 2003), and the overexpression effect (*ppe38*) (Beggs et al., 2000; Safi et al., 2004; Soto et al., 2004; Alonso et al., 2011) (**Figure 6**). No differences were detected in the transcription of *dnaA* or *dnaN* genes between case 0 and case 129. Second, we could observe a higher transcription of IS*6110* in the three MtZ isolates analyzed compared with H37Rv. This means that MtZ had some of its IS copies overexpressed. We speculate that it could be the one in *desA3*, as this gene has high levels of transcription, even higher in the stationary growth phase. Consequently, there will be a large number of IS*6110* reads mapped with all of the H37Rv ISs in the genome responsible for the high peaks we have observed.

According to other authors (Ates et al., 2018), PE_PGRS secretion is related to virulence. These authors have observed that deletion of the *ppe38/71* locus prevents this secretion from making Beijing strains hypervirulent. We demonstrated that MtZ, despite having both *ppe38/71* genes affected by an IS*6110* insertion, still secretes PE_PGRS proteins. The cause of having overexpressed the PE_PGRS proteins could be precisely the IS*6110* inserted in *ppe38*. Although IS*6110* is inserted within the gene, an ORF predictor showed the existence of an ORF in which the resulted protein was PPE38 lacking the first 40 amino acids. We hypothesize that this protein could conserve part of its functional activity. The possibility that IS*6110* could be acting as a promoter would be supported by the fact that the transcriptomic profile also showed overexpression of the *ppe38* gene in the exponential growth phase. It should be mentioned that in a previous work, we showed that an IS*6110* insertion upstream of the two-component regulator PhoP strongly increased the expression of this gene (Soto et al., 2004) and also had phenotypic consequences. The specific insertion of the IS*6110* in the herein observed case might have a similar function.

The study had some limitations. First, just 23.7% of the isolates belonging to the cluster were sequenced; therefore, several links and new transmission clusters have been lost. Because of this, we cannot know the true extent of CLS-2.2 and CLS-2.3, for which transmitted SNPs in genes related to pathogenesis were involved. Moreover, data for 1996–2000, when the strain probably started to spread with more force, are not available. The COVID-19 pandemic required that we perform the sequences with different platforms, making the analysis in BioNumerics software more difficult. This was solved by analyzing the SNPs detected in the isolates singularly.

In conclusion, the MtZ strain produced the largest outbreak ever reported in the Aragon region, and this outbreak remains active even today despite a general lack of risk factors for developing TB among the population. Fortunately, it is a drug-susceptible strain and patients can be cured with treatment. WGS allowed us to clarify the transmission chain more than epidemiological information alone although the direction of transmission was not solved for all cases. MtZ includes several SNPs in genes considered to be virulence factors as well as genes involved in pathogenesis and survival, which could be the cause of its success. More research is needed to know how the higher IS*6110* transcription and the oversecretion of PE_PGRS observed in the MtZ strain affect its virulence and pathogenesis.

## Data Availability Statement

The datasets presented in this study can be found in online repositories. The names of the repository/repositories and accession number(s) can be found below: https://www.ncbi.nlm.nih.gov/genbank/, SAMN23235155–SAMN23235211.

## Author Contributions

JC wrote the manuscript, contributed to the conception and design of the study, analyzed the data, and performed the laboratory work. JM and RB performed some laboratory work, wrote sections of the manuscript, and revised the submitted manuscript. IR and MZ-A performed the geographical part of the study and wrote sections of the manuscript. DI, JV, LT, JS, and M-JI provided epidemiological support. AC, JG-A and CK provided bioinformatic support. SS wrote the manuscript, contributed to the conception and design of the study, and was responsible for funding acquisition. All authors contributed to the article and approved the submitted version.

## Funding

This work was supported by the Carlos III Health Institute in the context of a grant (FIS18/0336) and JC was awarded a scholarship by the Government of Aragon/European Social Fund, “Building Europe from Aragon”. JM and RB acknowledge the support by the Agence Nationale de la Recherche (grants ANR-10-LABX-62-IBEID and ANR-20- CE15-0013-03).

## Conflict of Interest

The authors declare that the research was conducted in the absence of any commercial or financial relationships that could be construed as a potential conflict of interest.

## Publisher’s Note

All claims expressed in this article are solely those of the authors and do not necessarily represent those of their affiliated organizations, or those of the publisher, the editors and the reviewers. Any product that may be evaluated in this article, or claim that may be made by its manufacturer, is not guaranteed or endorsed by the publisher.
